# Specificity of inhibitory KIRs enables NK cells to detect changes in an altered peptide environment

**DOI:** 10.1007/s00251-017-1019-1

**Published:** 2017-07-10

**Authors:** Paola Carrillo-Bustamante, Rob J. de Boer, Can Keşmir

**Affiliations:** 1 Theoretical Biology and Bioinformatics, Department of Biology, Utrecht University, Utrecht, The Netherlands; 2 Center for Modeling and Simulation in the Biosciences (BIOMS/IWR), Max Planck Institute, Heidelberg, Germany

**Keywords:** KIR, NK cells, Viral infection, Peptide sensitivity

## Abstract

The activity of natural killer (NK) cells is tightly regulated by inhibitory and activating receptors. Inhibitory killer immunoglobulin-like receptors (iKIRs) survey the surface of target cells by monitoring the expression of human leukocyte antigen (HLA) class I. The binding of iKIRs has been shown to be sensitive to the peptides presented by HLA class I, implying that iKIRs have the ability to detect the changes in the repertoire of peptide-HLA class I complexes (pHLA), a process occurring during viral infection and in tumor cells. To study how the pHLA repertoire changes upon infection, and whether an iKIR is able to detect these changes, we study peptides eluted from cells prior and after infection with measles virus (MV). Remarkably, most changes in the repertoire of potential iKIR ligands are predicted to be caused by the altered expression of self-peptides. We show that an iKIR can detect these changes in the presented peptides only if it is sufficiently specific, e.g., if iKIRs can distinguish between different amino acids in the contact residues (e.g., position 7 and 8). Our analysis further indicates that one single iKIR per host is not sufficient to detect changes in the peptide repertoire, suggesting that a multigene family encoding for different iKIRs is required for successful peptide recognition.

**Electronic supplementary material:**

The online version of this article (doi:10.1007/s00251-017-1019-1) contains supplementary material, which is available to authorized users.

## Introduction

**Fig. 1:**
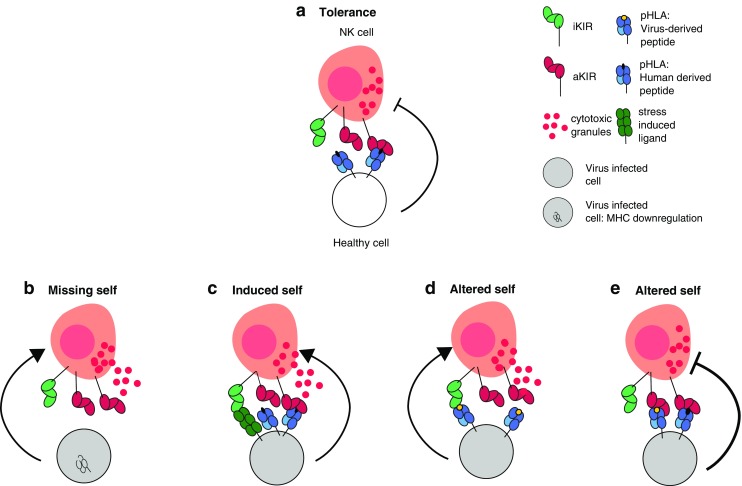
Models of NK cell activation: How can NK cells recognize virus infected or aberrant cells. The main ligands for iKIRs (depicted in *red*) in humans are HLA-I molecules (depicted in *blue*), which render NK cells tolerant towards healthy tissue (**a**). The current models for NK cell activation include “missing self detection” (**b**), and “induced self detection” (**c**), where virus-infected or malignant cells down-regulate the expression of HLA-I, or up-regulate stress ligands for activating KIRs (aKIRs, depicted in *green*), respectively. Complementing these models is “altered-self,” describing the sensitivity of iKIRs to changes in the peptide repertoire presented by HLA-I (**d**, **e**). A different peptide-HLA repertoire caused, e.g., by a virus-derived peptide (depicted in *yellow*) can promote aKIR binding or block iKIR binding resulting in enhanced NK cell activation (**d**). Alternatively, changes in the peptide repertoire (caused by, e.g., a virus-derived peptide) may enhance the binding of iKIRs to their HLA-I molecules, resulting in a stronger inhibiting signal (**e**)

Natural killer (NK) cells are key players of the innate immune response. Their activity is tightly regulated by several germline encoded inhibitory and activating receptors, including the highly polymorphic killer immunoglobulin-like receptors (KIRs) that interact with the classical major histocompatibility complex (MHC) class I molecules (in humans encoded by the HLA complex) expressed on target cells. Because inhibiting signals dominate over activating signals, healthy cells do not initiate NK cell cytotoxicity. However, virus-infected or malignant cells may up-regulate ligands for activating receptors (“induced self detection”) or down-regulate ligands for inhibitory receptors (“missing self detection”), thereby inducing NK cell activation and target cell lysis (Fig. 1) (Vivier et al. 2008; Lanier 2005).

Unlike T cell receptors, inhibitory KIRs (iKIRs) are not highly specific for particular peptide-HLA class I complexes (pHLA). Instead, they recognize subsets of class I HLA (HLA-I) molecules sharing structural motifs, including four mutually exclusive epitopes on HLA-I alleles: A3/11, Bw4, C1, and C2 (Moretta et al. 1996). Additionally, several studies have shown that iKIRs are also sensitive to the peptides bound by HLA-I molecules (Malnati et al. 1995; Rajagopalan and Long 1997; Peruzzi et al. 1996; Thananchai et al. 2007; Hansasuta et al. 2004; Fadda et al. 2010). Crystal structures of KIR2DL1 and KIR2DL2 in complex with their HLA-C ligands further supported this observation by revealing that specifically positions 7 (P7) and 8 (P8) of the HLA-I bound peptide are in direct contact with residues of inhibitory KIRs (Boyington et al. 2000; Brooks et al. 2000; Fan et al. 2001; Li and Mariuzza 2014. This suggests that NK cell activation may be regulated in a peptide dependent manner. Indeed, pHLA that have weak or no binding to iKIRs can efficiently reduce KIR-mediated inhibition (Cassidy et al. 2014; Fadda et al. 2010). Other studies also show that sequence variations within HLA-C restricted HIV epitopes have a large impact on the binding of inhibitory KIR2DL2, with some peptides enhancing and others disrupting the binding, and consequently abrogating the inhibition of NK cells (Fadda et al. 2012; Van Teijlingen et al. 2014). Also in the context of simian immunideficiency virus (SIV), new findings demonstrate how viral peptides modulate NK cell responses through KIR-MHC-I interactions, increasing the binding of MHC-I ligands to iKIRs as a mechanism to suppress NK cell responses (Schafer et al. 2015).

These studies convincingly underline potential functional consequences of the presented peptide in KIR-MHC-I interactions, and thereby in NK cell regulation. This calls for an extension of the current model of NK cell activation: “missing self” or “induced” (Fig. 1a, b) detection should be complemented by “altered self,” where changes in the MHC-I peptide repertoire after a viral infection modulate NK cell signaling (Fig. 1c, d). Depending on the repertoire of peptide-MHC-I complexes (pMHC), the KIR mediated activity of NK cells can be either promoted or inhibited. Stronger NK cell activation might arise if some pMHC bind activating KIRs, or if some pMHC disrupt the binding to iKIRs thereby abrogating inhibiting signals (Fig. 1c). Alternatively, strong NK cell inhibition would result from particular peptides enhancing the binding of iKIRs to MHC-I molecules (Fig. 1d). Indeed, viruses would escape from NK cell activation by encoding peptides increasing iKIR-pMHC binding (Alter et al. 2011; Schafer et al. 2015; Fadda et al. 2012; Van Teijlingen et al. 2014).

In this study, we will address two questions. First, is it likely that iKIRs can detect changes in the peptide repertoire presented by HLA-I molecules? And secondly, can this “peptide sensitivity” provide an explanation for evolution of specific iKIR molecules and thereby account for the high genetic diversity observed in the KIR family? To investigate the possibility of NK cell regulation via altered peptide repertoires, we analyze how the iKIR-ligands change after infection with measles virus (MV) using published peptide elution data from cells prior and after infection with this virus (Schellens et al. 2015a; Schellens et al. 2015b). Our analysis shows that iKIRs need to be specific to detect changes in the peptide repertoire, and that this required specificity might be sufficient to select for a multigene iKIR family.

## Results

### How do iKIR ligands change after a viral infection?

**Fig. 2:**
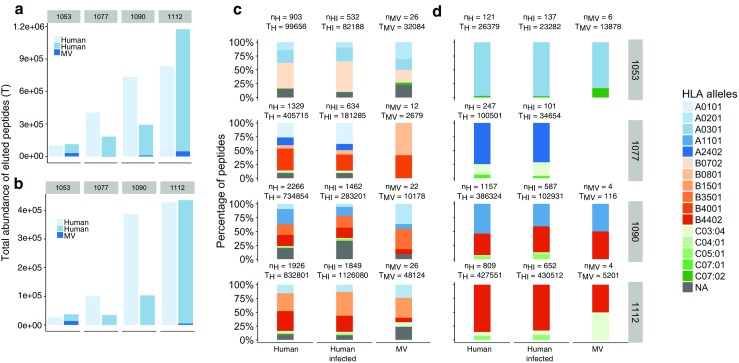
Distribution self and viral peptides in the four BLCL. We analyze the abundances of all eluted peptides (**a**, **c**) and those predicted to be restricted to HLA-I alleles expressing any of the four motifs A3/A11, Bw4, C1, and C2, i.e., potential ligands for iKIRs (**b**, **d**), before and after infection with MV. Given are the number unique peptides (*n*_*H*_, *n*_*HI*_, *n*_*MV*_), as well as their expression levels, i.e., abundances (*T*_*H*_, *T*_*I*_, *T*_*MV*_). **a**, **b** The *bars* depict the total abundance (T) of eluted human (self) peptides from uninfected (*T*_*H*_) and MV infected BLCLs (*T*_*HI*_), as well as the abundance of viral peptides (*T*_*MV*_). **c**, **d** We compute the fraction of eluted peptides that are predicted to bind to each particular HLA-I allele (*f*_*i*_ = nHL*A*_*i*_/*T*_∗_, where nHL*A*_*i*_ is the total abundance of eluted peptides predicted to bind to HL*A*_*i*_, and depending on the set of analyzed peptides, *T*_∗_ is *T*_*H*_, *T*_*HI*_,or*T*_*MV*_ (see “Material and methods” section). Assigned HLA-I alleles are given on the right (“NA” are peptides that could not be assigned to any HLA-I molecule). Data from Schellens et al. (2015a), Schellens et al. (2015b)

Although several studies have tried to address how the pHLA repertoire changes during a viral infection (Wahl et al. 2010; Rucevic et al. 2016; Ternette et al. 2016; Spencer et al. 2015), there are no quantitative estimates of how an infection affects iKIR ligands. To tackle this question, we made use of recent data of four B lymphoblastoid cell-lines (BLCLs) infected with MV (Schellens et al. 2015a; Schellens et al. 2015b). The details of this study are fully described in Schellens et al. (2015a), Schellens et al. (2015b). Briefly, pHLA were purified from four BLCLs, after which acid elution was applied to separate the peptides from the HLA-I molecules. All four BLCLs were either left uninfected, or infected with MV. To isolate the pHLA complexes, the authors used an antibody with a comparable affinity for all three major HLA class I molecules (HLA-A, -B and -C). As a result, the HLA-I assignment of the eluted peptides was unknown, and a peptide-HLA-I binding affinity prediction program (NetMHC 3.0 Lundegaard et al. 2008; Nielsen and Andreatta 2016) was used to determine which of the HLA class I molecules expressed by BLCLs was most likely presenting the identified peptides. The HLA-I molecules of BLCLs are given in Fig. 2. With this approach, the number of unique self peptides in uninfected and infected BLCLs (*n*_*H*_, *n*_*HI*_, respectively) and the number of unique viral peptides (*n*_*MV*_) associated to particular HLA-I alleles; as well as the concentration (i.e., total abundance (T)) of each unique peptide could be determined (Fig. 2).

There is a large variation in the number of presented peptides among the four different cell lines after the infection with MV. The number of unique peptides decreases in all BLCLs after infection (compare *n*_*H*_ to *n*_*HI*_ + *n*_*MV*_ in Fig. 2), with more than 50% of specific peptides completely disappearing (Table 1). However, the total abundance of peptides increases in BLCLs 1053 and 1112, and decreases in BLCLs 1090 and 1077 (compare *T*_*H*_ to *T*_*HI*_ + *T*_*MV*_ in Fig. 2a, b). Similarly, the presentation of peptides by HLA-alleles differs in each BLCL, and the identified peptides are not equally distributed over the expressed HLA class I molecules (Fig. 2c, d). Importantly, the majority of peptides were derived from self proteins, whereas only very few peptides were derived from viral proteins. We performed a second analysis of only those peptides that are presented by the HLA-I molecules carrying the A3/A11, Bw4, C1, or C2 epitope, since the main ligands for iKIRs include at least these four motifs (Trowsdale et al. 2001) (Fig. 2b). The distribution of this peptide subset is very similar to that of all eluted peptides, suggesting that the abundance of iKIRs ligands (hereafter referred to as *ligand density*) increased in two out of the four cell lines studied here, and decreased in the other two.

**Table 1:** Fraction of unique human peptides disappearing after MV infection. Summarized from Schellens et al. (2015a), Schellens et al. (2015b)

	BLCL identifier			
HLA-I alleles	1053	1077	1090	1112
All	0.66	0.74	0.73	0.59
A3/11, Bw4, C1, C2	0.59	0.74	0.72	0.61

We analyze the peptides predicted to be presented by all HLA-I molecules and those restricted to HLA-I alleles expressing any of the four motifs A3/A11, Bw4, C1, and C2, i.e., potential ligands for iKIRs. The fraction of unique peptides is defined as $\frac {n_{\mathrm {H}} \setminus n_{\text {HI}}}{n_{\mathrm {H}}}$, where *n*_H_ ∖ *n*_HI_ is the number of unique human peptides that is not found in the human peptides of MV-infected cells, and *n*_H_ the total number of unique human peptides eluted from uninfected BLCLs

### Sequence-based analysis of iKIRs ligands

The threshold for NK cell activation is determined during development, in a process called NK cell education or licensing (Bessoles et al. 2014; Höglund and Brodin 2010; Brodin et al. 2009). During this process, the amount of inhibitory and activating signals are balanced, preventing NK cells from targeting healthy tissue. Therefore, changes in any of the signals NK cell receive, e.g., upon viral infections or cancer, could disrupt this balance. The increased peptide/ligand density after MV infection suggests then that some iKIRs could receive an even stronger inhibiting signal, hampering the activation of those NK cells expressing that iKIR. Note however that different NK cells expressing different iKIRs will recognize different subsets from all these peptides. For any iKIR detecting a decrease in its set of ligands, all NK cells carrying this iKIR would receive a reduced inhibiting signal and could become activated. Thus, the probability of a single iKIR detecting a decrease of ligands should determine whether or not there will be quantitative change inx NK cell response.

**Fig. 3:**
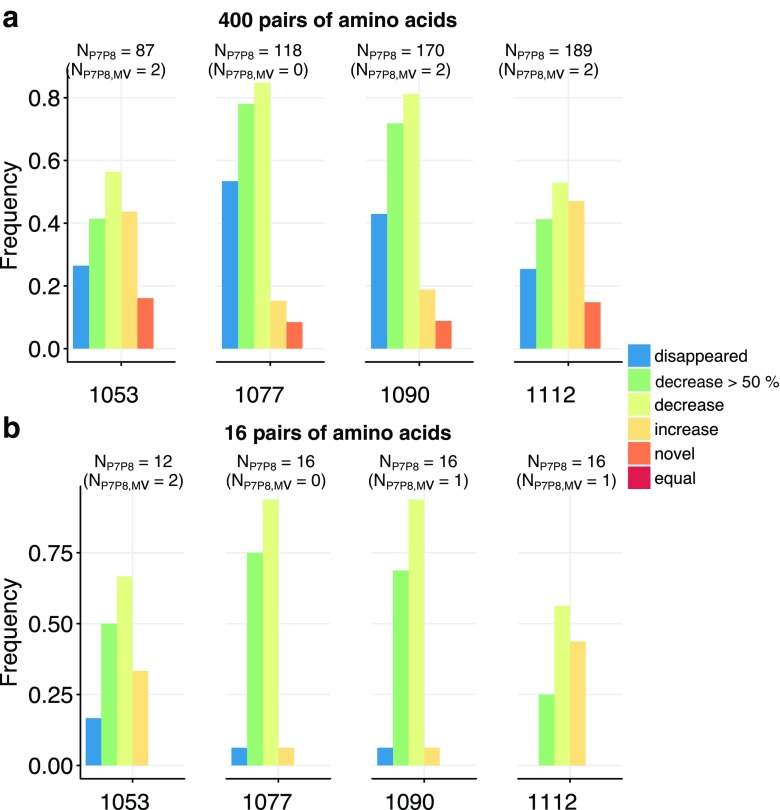
Changes in the total abundance of motifs for specific iKIRs using the sequence based analysis. HLA-I eluted 9-mers (restricted to HLA-I molecules carrying the A3/A11, Bw4, C1, or C2 alleles) were grouped according to (**a**) their unique amino acid combinations in P7 and P8 (i.e., assuming 400 different pairs of amino acids), or (**b**) into four distinct groups based on their physico-chemical properties (i.e., 16 different pairs of amino acids). The number of unique KIR motifs (i.e., unique amino acid pairs, or pairs of amino acids groups in P7 and P8) is given by *N*_*P*7*P*8_, out of which *N*_*P*7*P*8,*MV*_ motifs are derived from viral peptides. In this analysis, we monitor for each iKIR motif whether its abundance increases (*yellow*), or a decreases (*lime green*) after infection with MV. Using the percentual change, we also quantify the changes in ligand density after MV infection. The frequency of decreases by at least 50% is depicted in *dark green* and the loss in ligands in blue. Novel ligands, i.e., peptides carrying the KIR binding motifs present only in infected cells are represented in *orange*

To study “altered-self” recognition, we performed a sequence based analysis of the eluted peptides before and after the infection with MV. First, we analyzed the 9-mers among the peptides presented by HLAs carrying either the A3/A11, Bw4, C1, or C2 motif. Given that iKIRs are in contact with P7 and P8 of the presented peptide (Boyington et al. 2000; Brooks et al. 2000; Fan et al. 2001; Li and Mariuzza 2014) and that there can be 20 amino acids per position, we first assumed that iKIRs are able to distinguish between any of the 400 pairs of amino acids in those residues (i.e., a maximal specificity of iKIRs) and grouped the peptides according to the unique amino acid combinations in P7 and P8 (for a detailed description see “Material and methods” section). To determine the density of iKIR ligands, we summed the abundances of all peptides carrying these “KIR motifs.” We then counted how often there is a decrease, or an increase, in the ligand density after MV infection, thereby estimating how likely an individual iKIR can detect changes in its pool of ligands. The results are summarized in Fig. 3.

In all BLCLs, the number of KIR motifs (N_P7*P*8_ in Fig. 3) is smaller than the theoretical maximum of 400, because not all possible amino acid pairs are present in P7 and P8 of the peptides presented by these cell lines . The majority of motifs are derived from self-peptides, confirming that most changes in the ligand pool are not due to viral proteins (particulary, in BLCL 1077 no viral peptides were associated with any of the HLA-I molecules that is a traditional iKIR ligand, i.e., N_P7*P*8,*MV*_ = 0). The density of KIR ligands changes remarkably after infection. In all BLCLs, infection with MV results in up-regulation of some iKIR ligands, including novel peptides which are only expressed in infected cells (Fig. 3a, orange bars). However, infection with MV causes a decrease in ligand density for most iKIRs (Fig. 3a, light green bars) in *all* cell lines; a surprising observation given that BLCL 1112 and 1053 have an enhanced peptide presentation. The most striking case is BLCL 1112, in which almost 50% of the iKIRs are expected to detect a decrease in their ligands.

Because iKIRs are not merely sensitive to the presence of a peptide but also to the total abundance of pHLA complexes they interact with (reviewed in Cassidy et al. 2014), it is important to quantify by how much the abundance of KIR motifs changes after viral infection, and thereby predict the functional consequences of “altered-self” recognition. We indeed observe that the density of a large proportion of KIR ligands decreases by at least 50% (Fig. 3a, dark green bars), and some iKIR ligands completely disappear in all cell lines (Fig. 3a, blue bars). Since degranulation of NK cells increases linearly with decreasing ligand concentration (Cassidy et al. 2014), the strong decrease in KIR motifs observed here is likely to induce a strong NK cell activation. Thus, if highly specific, a large proportion of iKIRs are expected to detect substantial changes in the peptides presented by the MV infected BLCLs studied here.

Until now, we have considered maximal specificity of iKIRs, i.e., we assumed that they can discriminate between any pair of amino acids in P7 and P8. iKIRs are probably less specific and could, for instance, just recognize groups of amino acids based on their physico-chemical properties (e.g., non polar, polar, basic and acidic) in P7 and P8. If there were four such amino acid groups, the chance of any peptide becoming an iKIR ligand would increase. Our results hardly change after decreasing the specificity (Fig. 3b). Although the frequency of disappearing ligands is reduced, subsets of iKIRs are still able to detect decreases in their binding motif. Altogether, our sequence based analysis suggests that sufficiently specific iKIRs are expected to detect a decrease, or even loss, in their ligands in all four BLCLs studied here.

### iKIR can detect decreases in ligand concentration provided they are sufficiently specific

**Fig. 4:**
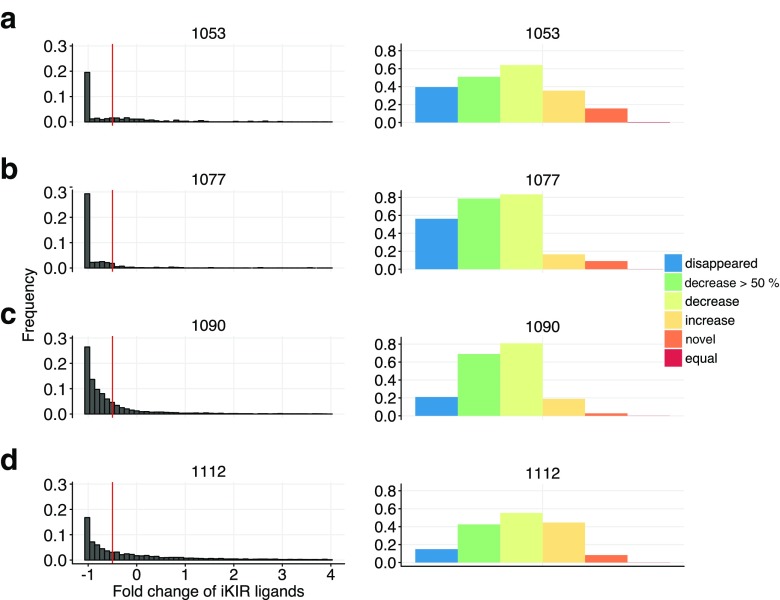
Distribution of iKIR ligands in four BLCLs using a sampling model. The group of peptides forming the set of iKIR ligands is determined by sampling with probability *p* = 1/400 from the pool of n = *n*_*H*_ + *n*_*HI*_ + *n*_*MV*_ unique peptides in BLCL 1053 (**a**), 1077 (**b**), 1090 (**c**), and 1112 (**d**). The distribution of changes in ligand density after sampling 10,000 times is depicted in the *right column*. The fold change is calculated as (*L*_*I*_ − *L*_*U*_)/*L*_*U*_, where *L*_*U*_ and *L*_*I*_ are the ligand densities in uninfected and infected cells, respectively (see “Material and methods”). Visualized are fold-changes with a frequency larger than 1%. The *red vertical line* shows decreases of 50%. On the *right column*, the *bars* depict the total occurrence of having an increased (*yellow*), decreased (*lime green*), or equal (*red*) ligand concentration after MV infection. Decreases by at least 50% are depicted in *dark green* and the loss in ligands in *blue*. Novel ligands, i.e., peptides present only in infected cells are represented in *orange*

Because we do not precisely know how iKIRs recognize their ligands we also analyzed the data using a more general sampling model where iKIRs bind any peptide with a pre-defined probability *p* (see “Material and methods” section). Similar to the sequence-based analysis, we determined the ligand density for a single iKIR by adding the abundance of each sampled peptide. After each sample, we compared whether the ligand density obtained by sampling uninfected cells is larger, or smaller, than that obtained by sampling after infected cells (Fig. 4). Repeating this procedure 10,000 times for all four BLCLs allowed us to generalize the findings from the sequence-based analysis, and robustly estimate the chance of one random iKIR detecting a decrease of ligands after the infection (Fig. 4).

We started by setting *p* = 1/400, which would correspond to the maximal specificity used above. Even though we here ignored the identity of the positions recognized by iKIRs, we found similar results (Fig. 4). The density of iKIR ligands typically decreases in BLCLs having a decreased peptide presentation after infection, i.e., BLCL 1090 and 1077 (lime green bars Fig. 4b, c). In these cell lines, at least 80% of the “simulated” iKIRs detect a lower density of ligands. Interestingly, we also found that subsets of iKIRs can detect a decrease in their ligands even when peptide presentation is enhanced after MV infection (i.e., BLCLs 1053 and 1112, Fig. 4a, d). Once again, BLCL 1112 is particularly interesting, as more than half of the simulated iKIRs are expected to detect a decrease in their ligands, 40% of which recognize a decrease of at least half and approximately 15% detect a loss (Fig. 4d). The strong decrease in ligand density predicted by our sampling model implies a potential strong NK cell activation in all cell lines.

**Fig. 5:**
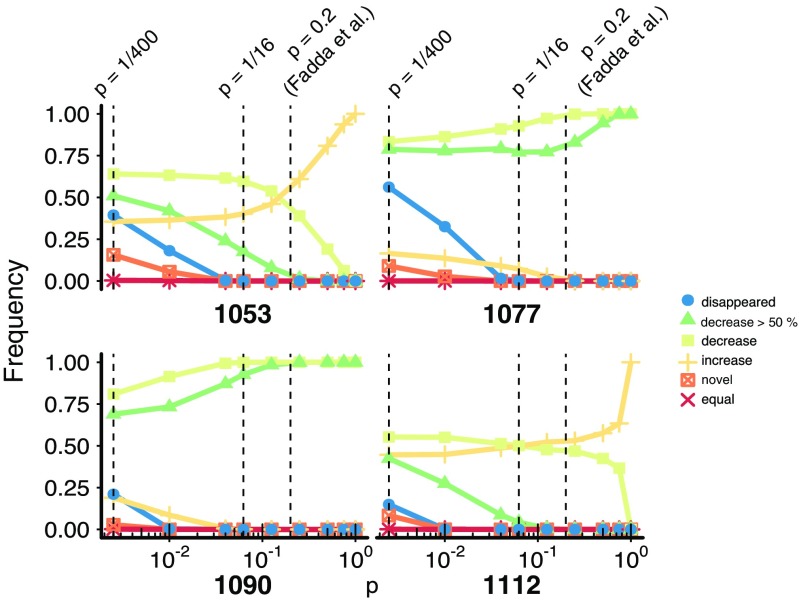
Changes in the iKIR binding repertoire as a function of iKIRspecificity. Probability of a random iKIR detecting “altered-self” given as the frequency of iKIR ligand densities that were predicted by our sampling model to change after infection. To quantify how the specificity influences the changes in ligand density, the model was run for various values of *p*: 0.0025, 0.01, 0.04, 0.0625, 0.1250, 0.25, 0.50, 0.7500, and 1. Highlighted with the *dashed black lines* are the values *p* = 1/400, *p* = 1/16, *p* = 0.2. An iKIR successfully detects “altered-self” if it recognizes a decrease in its ligands, with larger functional consequences if the detected decrease is larger than 50%. The changes in ligand density are depicted in the *rainbow scale*

To quantify how iKIR specificity relates to their capacity to detect changes in ligand densities, we repeated this sampling analysis for various values of *p* (Fig. 5). Less specific iKIRs (which would correspond to *p* = 1/16) are less likely to recognize substantial decreases in cells with an increased peptide presentation (i.e., BLCL 1053, and 1112; Fig. 5). To have a more accurate estimate of the actual iKIRs specificity, we adopted data published by Fadda et al. (2010), where the binding of KIR2DL2, KIR2DL3, and KIR2DS2 to HLA-C-peptide complexes was studied. Out of 59 peptides where amino acids in P7 and P8 form a unique pair, 13 were described as KIR2DL2 binders (Supplementary Table 1 in Fadda et al. 2010), suggesting that the probability of this iKIR recognizing a peptide as a ligand is approximately *p* = 0.2. Also in this specificity range, most iKIRs are unable to recognize large decreases in their ligands in cells with enhanced peptide presentation (i.e., presenting a larger abundance of peptides, Fig. 5). In BLCL 1053, less than 10% of the sampled iKIRs detect a large depletion of their ligands, whereas none of the iKIRs is able to detect these substantial reductions in BLCL 1112.

Our model shows that the more degenerate iKIRs are (i.e., the larger *p*), the more challenging it is for them to detect disappearing ligands (Fig. 5). In fact, if all iKIRs are completely degenerate (i.e., *p* = 1), they can only detect large decreases in their binding repertoire in cells having reduced peptide presentation after MV infection (BLCL 1077 and 1090 in Fig. 5). However, in cells with increased peptide presentation (i.e., 1053 and 1112), subsets of iKIRs can detect reductions of their ligands only if they are highly specific (i.e., with *p* < 0.1, blue line in Fig. 5). Our model thus shows that successful detection of “altered-self” in *all* four cell lines examined can only be achieved if iKIRs are more specific than estimated by Fadda et al. (2010).

### Specific recognition of peptides requires several iKIRs

Having an estimation of the probability that an iKIR recognizes changes in the ligand pool from both our data analysis and sampling model (Figs. 3, and 5), we can speculate about the number of receptors that an individual host would require for a successful iKIR mediated NK cell response.

Consider a “worst-case” scenario, e.g., BLCL 1112 in Fig. 5. After interacting with this cell line, less than 15% of highly specific iKIRs (*p* = 1/400) would result in a strong NK cell activation, as less than 15% of KIR motifs disappear upon the viral infection (BLCL 1112 in Fig. 5). Because an individual basically needs at least one iKIR detecting altered self (as all NK cells expressing that iKIRshould become activated and respond), it is tempting to speculate that six, or seven, different highly specific iKIRs per individual would be sufficient to successfully detect “altered-self” in BLCL 1112. Note that NK cell degranulation increases linearly when the ligand density (i.e. concentration of pHLA complexes) on the cell surface decreases (Cassidy et al. 2014). If a reduction of iKIR ligands at least by half were necessary to mount a successful NK cell response, approximately 40% of iKIRs in BLCL 1112 would detect a decrease in their ligands (Fig. 5), implying that two to three iKIRs would be required for NK cell activation.

To have a lower estimate for the required KIR diversity, consider now a “best-case scenario” (e.g., BLCL 1077 in Fig. 5). Here, more than 50% of iKIRs would completely lose their ligands (BLCL 1077 in Fig. 5), and 80% of iKIRs would detect ligands decreasing in their abundance by half (BLCL 1077 in Fig. 4b). Thus, two iKIRs would be sufficient to detect a decrease in ligands, and result in activation of NK cells expressing these iKIRs. This analysis of the best and worst case scenarios indicate that one single iKIR is not sufficient for “altered-self” detection.

## Discussion

The potential functional consequences of the presented peptide in KIR-HLA interactions have remained unresolved. Here, we use data analysis and a simple modeling approach to study (1) how the peptide repertoire changes after a viral infection, and (2) the probability of iKIRs to recognize these changes. We show that most changes in the peptide pool are originating from altered presentation of self-proteins (probably due to gene expression changes), and that iKIRs can detect decreases in the peptide pool if they are sufficiently specific.

The importance of peptide sensitivity for iKIRs has been mostly related to viral infections, especially in HIV-1 infections (Fadda et al. 2012; Van Teijlingen et al. 2014). Recent studies demonstrate that sequence variations within HLA-C restricted HIV epitopes strongly engage inhibitory KIR2DL2, inducing a strong inhibiting signal for NK cells (Van Teijlingen et al. 2014). Although these studies certainly have important functional implications, especially regarding viral escape mechanisms, our study shows that changes in the presented self-peptides can be sufficient to detect a viral infection. If the HIV-1 peptides are not highly abundant on productively infected cells, their contribution to the total changes in the peptide repertoire might be small. Indeed, all changes in the peptide repertoire (i.e., derived from self and viral proteins) as well as the abundance of the peptides are necessary to estimate the implications of peptides on iKIR mediated NK cell regulation.

The BLCL data set allowed us to investigate how the peptide pool that is relevant for iKIR-binding (i.e., the peptides restricted to HLA-A3/A11, -Bw4, C1, and C2) changes after a viral infection. A crucial parameter in all our analyses is the total abundance of peptides, which was estimated by comparing the mass-spectrometry (MS) response of two specific standard peptides to those eluted from the BLCLs (Schellens et al. 2015a; Schellens et al. 2015b). It should be noted that this type of quantification is based on the assumption that the eluted and standard peptides have equal response factors (count/mole) in the MS analysis, and that not all peptides behave equally. Hence, the abundances might not be accurate. Additionally, it is important to note that the assignment of the eluted peptides to the HLA alleles was predicted *in silico*, and possibly some eluted peptides were not assigned to any HLA molecule. Furthermore, the HLA-C restricted peptide pool might have been underestimated, as there is a preference in assignment to HLA-A and -B alleles, because the prediction performance for the better-studied HLA-A and HLA-B alleles is higher than that for HLA-C alleles. Even if the contribution of these “possible ligands” is underestimated, the main outcome of our study does not change much, as the analysis including all 9-mers (even considering also peptides that have not been assigned to any HLA molecule) show results very similar to those considering only the group of peptides presented by HLA-A3/A11, -Bw4, C1, and C2 (Fig. S1).

By considering that an iKIR can discriminate any pair of amino acids in P7 and P8, we assumed the maximal iKIRspecificity (i.e., *p* = 1/400). However, neither in the data set of Schellens at al., nor in that from Fadda et al., all 400 pairs of amino acids are present among the peptides studied. It is indeed likely that not all pairs of amino acids are presented by the HLA molecules studied in those studies, or that some combinations do not frequently occur. The actual iKIR specificity is necessary to make a proper conclusion about diversity. In our sampling model, we show that the required genetic KIR diversity varies widely among BLCLs, with approximately six or seven specific iKIRs being necessary to detect changes in the peptide repertoire in BLCL 1112, and two iKIRs being sufficient in BLCL 1077 if iKIRs can distinguish between all 400 duplets in P7 and P8 of a pHLA. If the real specificity were lower, like suggested by Fadda’s study (where 13/59 peptides were KIR-binders Fadda et al. 2010), the detection of the altered peptide repertoire would become more challenging, as shown by our sampling model (Fig. 5). Similarly, if iKIRs were to recognize groups of amino acids in all presented 9-mers, the detection of altered-self would be hampered (Figs. 3b and 5). Note that by restricting our model and sequence analysis to the peptides presented by HLA-I alleles relevant for iKIR binding (i.e., HLA-A3/A11, Bw4, C1, and C2), we have ignored an additional element of specificity. In reality, iKIRs must first bind to one of these alleles, and then be specific for the binding motifs in the peptide. Since this increases the specificity, even more iKIRs per individual would be necessary to detect the viral infection.

To our knowledge, this is the first study that comprises a detailed analysis of the variation in the iKIR-ligands after a viral infection. By combining a mathematical model with experimental data, we were able to estimate the numbers and direction of change in the peptide repertoire, shedding light on the complex mechanisms of peptide sensitivity and its possible functional consequences. Although the required number of iKIRs we have estimated in this study (six to seven in the “worst-case” scenario) is in line with the observed number of iKIRs in humans (Gendzekhadze et al. 2009; Wilson et al. 2000; Witt et al. 1999), further studies are indispensable to get a better estimate about the genetic diversity and optimal specificity of iKIRs. To this end, we have previously studied the evolution of KIRs by using an evolutionary computational model (Carrillo-Bustamante et al. 2013, 2014, 2015a, b). By expanding those models with the understanding of the peptide repertoire prior and after infection we have gained in this current study, we can more accurately study the selection pressure on iKIRs caused by peptide sensitivity.

## Material and methods

### Elution data

The details of the experimental setup are fully described in the studies of Schellens et al. (2015a). Briefly, HLA class I molecules were immunoprecipitated from uninfected and 48 h MV-infected BLCLs using the HLA-I specific monoclonal antibody W6/32. The peptide repertoires eluted from the HLA-I molecules were fractionated, and analyzed by high-resolution nanoscale liquid chromatography-mass spectrometry (nano-LC-MS) and mass sequencing. To identify the identity of the eluted peptides, the mass spectrometry (MS) data were processed using BioWorks (version 3.3.1 SP1; Thermo Scientific, San Jose, USA) against the human- and MV-annotated proteins extracted from the UniProtKB/Swiss-Prot database. The peptide expression levels (i.e., total abundance T) was determined by comparing their MS response to that of two standard peptides. This quantification procedure is based on the assumption of equal response factors (counts/mole) for the eluted peptides and these standard peptides in the MS analysis. By this approach, the number of unique peptides (n_H_,n_HI_,n_MV_) and the total abundance (T_H_,T_HI_,T_MV_) was determined.

Because the antibody W6/32 has comparable affinity for all three HLA-I molecules, the HLA-I assignment of the identified peptides was unknown, and a peptide-HLA-I binding affinity prediction tool (NetMHC3.0 Lundegaard et al. 2008; Nielsen and Andreatta 2016) was used to determine which of HLA-I molecules expressed by the cell lines were most likely to present the identified peptides. All identified peptide sequences, with their amino acid length, estimated total abundance, and the predicted HLA-I molecule are given in Supplementary Table 1 in Schellens et al. (2015a).

### Data analysis

For each set of peptides (i.e., self peptides eluted from uninfected and infected cells, and virus-derived peptides), the fraction of eluted peptides predicted to bind particular HLA-I allele was computed as *f* *i* = nHL*A*_*i*_/*T*_∗_, where nHL*A*_*i*_ is the total abundance of eluted peptides predicted to bind to HL*A*_*i*_, and *T*_∗_ is *T*_*H*_, *T*_*HI*_,or*T*_*MV*_ (depending on the set of peptides).

The proportion of disappearing peptides was calculated as $\frac {n_{\mathrm {H}} \setminus n_{\text {HI}}}{n_{\mathrm {H}}}$, where *n*_H_ and *n*_HI_ represent the number of unique self peptides eluted from uninfected and MV-infected cells, respectively. The term *n*_H_ ∖ *n*_HI_ denotes the number of unique self peptides found in uninfected cells that are not present in MV infected BLCLs.

### Sequence-based analysis

We extracted all 9-mers which had been associated to the HLA-I molecules carrying the A3/A11-Bw4, C1, or C2 epitope. To characterize “KIR motifs,” we grouped the selected peptides according to (1) the unique amino acid combinations and (2) four distinct amino acid groups in P7 and P8 (non polar, including Glycine, Alanine, Valine, Leucine, Isoleucine, Methionine, Phenylalanine, Tryptophan, and Proline; polar including Serine, Threonine, Cysteine, Tyrosine, Asparagine, and Glutamine; basic including Lysine, Arginine, and Histidine; and acidic including Aspartate and Glutamate). We then summed the abundances of all peptides carrying each of the *N*_*P*7*P*8_ unique KIR motifs, thereby determining the iKIR ligand density. Note that *N*_*P*7*P*8_ is composed of human-derived motifs in uninfected and infected cells (*N*_*P*7*P*8,*H*_ and *N*_*P*7*P*8,*HI*_), and MV-derived motifs (*N*_*P*7*P*8,*MV*_).

To analyze how the iKIR ligand density changes after MV infection, we monitored how often there was a decrease, or an increase in ligand abundance, in MV-infected BLCLs compared to uninfected cells. The changes in ligand density were categorized into three main groups: “decrease,” “equal,” and “increase.” Decreased ligand densities were additionally sub-categorized into “decrease > 50*%*” and “disappeared.” Similarly, “novel” ligands, i.e., KIR motifs present only in infected cells, were included in the “increase” category.

### Sampling model

We developed a simple sampling model that randomly selects a set of peptides as iKIR ligands and monitors how their density changes after MV-infection. We first defined *p* as the probability of a peptide being an iKIR ligand. Based on this probability value, we randomly selected peptides out of the *n* = *n*_*H*_ + *n*_*I*_ + *n*_*MV*_ unique peptides eluted from each BLCL. The expected number of unique iKIR ligands for a particular cell line (i.e., the number of “sampled” peptides) would then be *N* = *p* ×n. Next, we estimated the ligand density L for one iKIR by summing the measured abundances of each of the sampled peptides in uninfected (i.e., *T*_*H*_) and infected cells (*T*_*HI*_ + *T*_*MV*_). Thus, the ligand density in uninfected and infected cells is given by $\mathrm {L_{U}} = {\sum }_{i = 0}^{i =N} \mathrm {T_{H,i}}$, and $\mathrm {L_{I}} = {\sum }_{i = 0}^{i =N} \mathrm {T_{HI,i}} + {\sum }_{i = 0}^{i =N} \mathrm {T_{MV,i}}$, respectively. We quantified the change in ligand density for one iKIR by calculating the fold change (*L*_*I*_ − *L*_*U*_)/*L*_*U*_.

For every cell line, we repeated this procedure 10,000 times, obtaining a distribution of the fold change in ligand density (as shown in the left column of Fig. 4). Similar to the sequence-based analysis, we categorized the estimated fold change into three major categories: “decrease,” “equal,” and “increase.” Decreased ligand densities were additionally sub-categorized into “decrease > 50*%*” and “disappeared.” Similarly, “novel” ligands, i.e., peptides present only in infected cells, were included in the “increase” category (right column of Fig. 4).

For the analysis of peptides restricted to HLA-I molecules carrying one of the A3/A11, Bw4, C1, and C2 alleles, we used the number of unique peptides *n*_*H*_, *n*_*HI*_, *n*_*MV*_ as depicted in Fig. 2d. Accordingly, for the analysis including all peptides, we used the number of unique peptides *n*_*H*_, *n*_*HI*_, *n*_*MV*_ as depicted in Fig. 2c. To quantify how the specificity influences the changes in ligand density, we repeated this sampling for various values of *p*: 0.0025, 0.01, 0.04, 0.0625, 0.1250, 0.25, 0.50, 0.7500, and 1.

We used R scripts to analyse the data and perform the sequence-based analysis and sampling model. The source code is available upon request.

## Electronic supplementary material

Below is the link to the electronic supplementary material.

Figure S1. Changes in the total abundance of motifs for specific iKIRs using the sequence based analysis. All HLA-I eluted 9-mers were grouped according to (A) their unique amino acid combinations in P7 and P8 (i.e. assuming the maximal iKIR specificity), or (B) into four distinct groups based on their physico-chemical properties. The number of unique KIR motifs (i.e., unique amino acid pairs, or pairs of amino acids groups in P7 and P8) are given by N_P7P8_, out of which N_P7P8;MV_ motifs are derived from viral peptides. In this analysis, we monitor for each iKIR motif whether its abundance increases (yellow), or a decreases (lime green) after infection with MV. Using the percentual change, we also quantify the changes in ligand density after MV infection. The frequency of decreases by at least 50% is depicted in dark green and the loss in ligands in blue. Novel ligands, i.e., peptides carrying the KIR binding motifs present only in infected cells are represented in orange. (PDF 90.5 KB)

## References

[CR1] Alter G, Heckerman D, Schneidewind A, Fadda L, Kadie CM (2011). HIV-1 adaptation to NK-cell-mediated immune pressure. Nature.

[CR2] Bessoles S, Grandclément C, Alari-Pahissa E, Gehrig J, Jeevan-Raj B (2014). Adaptations of natural killer cells to self-MHC class I. Front Immunol.

[CR3] Boyington JC, Motyka SA, Schuck P, Brooks AG, Sun PD (2000). Crystal structure of an NK cell immunoglobulin-like receptor in complex with its class I MHC ligand. Nature.

[CR4] Brodin P, Kärre K, Höglund P (2009). NK cell education: not an on-off switch but a tunable rheostat. Trends Immunol.

[CR5] Brooks AG, Boyington JC, Sun PD (2000). Natural killer cell recognition of HLA class I molecules. Rev Immunogenet.

[CR6] Carrillo-Bustamante P, Keşmir C, de Boer RJ (2013). Virus encoded MHC-like decoys diversify the inhibitory KIR repertoire. PLoS Comput Biol.

[CR7] Carrillo-Bustamante P, Keşmir C, de Boer RJ (2014). Quantifying the Protection of Activating and Inhibiting NK Cell Receptors during Infection with a CMV-Like Virus. Front Immunol.

[CR8] Carrillo-Bustamante P, Keşmir C, De Boer RJ (2015a) A co-evolutionary arms race between hosts and viruses drives polymorphism and polygenicity of NK cell receptors. Molecular biology and evolution: msv096 10.1093/molbev/msv096 PMC4833080 25911231

[CR9] Carrillo-Bustamante P, Keşmir C, De Boer RJ (2015). Can selective MHC downregulation explain the specificity and genetic diversity of NK cell receptors?. Front Immunol.

[CR10] Cassidy SA, Cheent KS, Khakoo SI (2014). Effects of Peptide on NK cell-mediated MHC I recognition. Front Immunol.

[CR11] Fadda L, Borhis G, Ahmed P, Cheent K, Pageon SV (2010). Peptide antagonism as a mechanism for NK cell activation. Proc Natl Acad Sci U S A.

[CR12] Fadda L, Körner C, Kumar S, van Teijlingen NH, Piechocka-Trocha A (2012). HLA-Cw*0102-restricted HIV-1 p24 epitope variants can modulate the binding of the inhibitory KIR2DL2 receptor and primary NK cell function. PLoS Pathog.

[CR13] Fan QR, Long EO, Wiley DC (2001). Crystal structure of the human natural killer cell inhibitory receptor KIR2DL1-HLA-Cw4 complex. Nat Immunol.

[CR14] Gendzekhadze K, Norman PJ, Abi-Rached L, Graef T, Moesta AK (2009). Co-evolution of KIR2DL3 with HLA-C in a human population retaining minimal essential diversity of KIR and HLA class I ligands. Proc Natl Acad Sci U S A.

[CR15] Hansasuta P, Dong T, Thananchai H, Weekes M, Willberg C (2004). Recognition of HLA-A3 and HLA-A11 by KIR3DL2 is peptide-specific. Eur J Immunol.

[CR16] Höglund P, Brodin P (2010). Current perspectives of natural killer cell education by MHC class I molecules. Nat Rev Immunol.

[CR17] Lanier LL (2005). NK cell recognition. Annu Rev Immunol.

[CR18] Li Y, Mariuzza RA (2014). Structural basis for recognition of cellular and viral ligands by NK cell receptors. Front Immunol.

[CR19] Lundegaard C, Lund O, Nielsen M (2008). Accurate approximation method for prediction of class I MHC affinities for peptides of length 8, 10 and 11 using prediction tools trained on 9mers. Bioinformatics.

[CR20] Malnati MS, Peruzzi M, Parker KC, Biddison WE, Ciccone E (1995). Peptide specificity in the recognition of MHC class I by natural killer cell clones. Science.

[CR21] Moretta A, Bottino C, Vitale M, Pende D, Biassoni R (1996). Receptors for HLA class-I molecules in human natural killer cells. Annu Rev Immunol.

[CR22] Nielsen M, Andreatta M (2016). NetMHCpan-3.0; improved prediction of binding to MHC class I molecules integrating information from multiple receptor and peptide length datasets. Genome Med.

[CR23] Peruzzi M, Wagtmann N, Long EO (1996). A p70 killer cell inhibitory receptor specific for several HLA-B allotypes discriminates among peptides bound to HLA-B*2705. J Exp Med.

[CR24] Rajagopalan S, Long EO (1997). The direct binding of a p58 killer cell inhibitory receptor to human histocompatibility leukocyte antigen (HLA)-Cw4 exhibits peptide selectivity. J Exp Med.

[CR25] Rucevic M, Kourjian G, Boucau J, Blatnik R, Bertran WG (2016). Analysis of major histocompatibility complex-bound HIV peptides identified from various cell types reveals common nested peptides and novel T cell responses. J Virol.

[CR26] Schafer JL, Ries M, Guha N, Connole M, Colantonio AD (2015). Suppression of a natural killer cell response by simian immunodeficiency virus peptides. PLoS Pathog.

[CR27] Schellens IM, Hoof I, Meiring HD, Spijkers SN, Poelen MC (2015). Comprehensive analysis of the naturally processed peptide repertoire: differences between HLA-A and B in the immunopeptidome. PloS one.

[CR28] Schellens IM, Meiring HD, Hoof I, Spijkers SN, Poelen MC et al (2015b) Measles virus epitope presentation by HLA: novel insights into epitope selection, dominance, and microvariation. Frontiers in Immunology 6 10.3389/fimmu.2015.00546 PMC4629467 26579122

[CR29] Spencer CT, Bezbradica JS, Ramos MG, Arico CD, Conant SB (2015). Viral infection causes a shift in the self peptide repertoire presented by human MHC class I molecules. Proteomics-Clin Appl.

[CR30] Ternette N, Yang H, Partridge T, Llano A, Cedeño S (2016). Defining the HLA class I-associated viral antigen repertoire from HIV-1-infected human cells. Eur J Immunol.

[CR31] Thananchai H, Gillespie G, Martin MP, Bashirova A, Yawata N (2007). Cutting Edge: Allele-specific and peptide-dependent interactions between KIR3DL1 and HLA-A and HLA-B. J Immunol.

[CR32] Trowsdale J, Barten R, Haude A, Stewart CA, Beck S (2001). The genomic context of natural killer receptor extended gene families. Immunol Rev.

[CR33] Van Teijlingen NH, Hölzemer A, Körner C, García-Beltrán WF, Schafer JL (2014). Sequence variations in HIV-1 p24 Gag-derived epitopes can alter binding of KIR2DL2 to HLA-C*03: 04 and modulate primary natural killer cell function. AIDS.

[CR34] Vivier E, Tomasello E, Baratin M, Walzer T, Ugolini S (2008). Functions of natural killer cells. Nat Immunol.

[CR35] Wahl A, Schafer F, Bardet W, Hildebrand WH (2010). HLA class I molecules reflect an altered host proteome after influenza virus infection. Hum Immunol.

[CR36] Wilson MJ, Torkar M, Haude A, Milne S, Jones T (2000). Plasticity in the organization and sequences of human KIR/ILT gene families. Proc Nat Acad Sci.

[CR37] Witt CS, Dewing C, Sayer DC, Uhrberg M, Parham P (1999). Population frequencies and putative haplotypes of the killer cell immuniglobulin-like receptor sequences and evidence for recombination. Transplantation.

