# Supplementary figures and images for: Specificity of inhibitory KIRs enables NK cells to detect changes in an altered peptide environment
Source: Immunogenetics. 2017 Jul 10;70(2):87–97. doi: 10.1007/s00251-017-1019-1 (PMC5775373; doi:10.1007/s00251-017-1019-1)

## 400 pairs of amino acids

**A**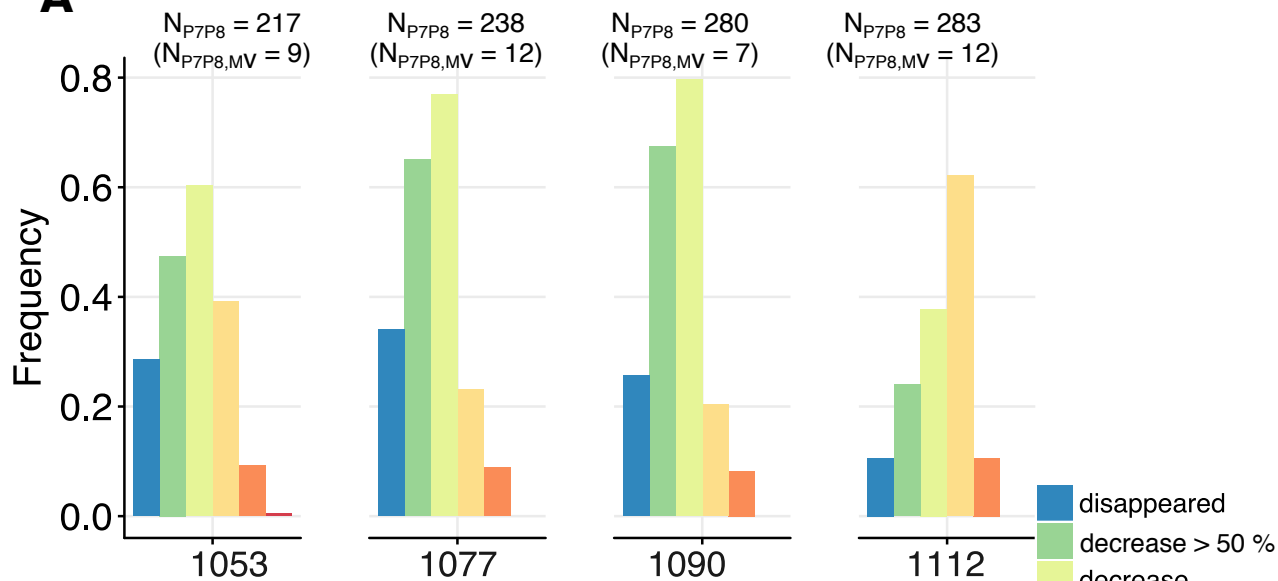**B**

## 16 pairs of amino acids

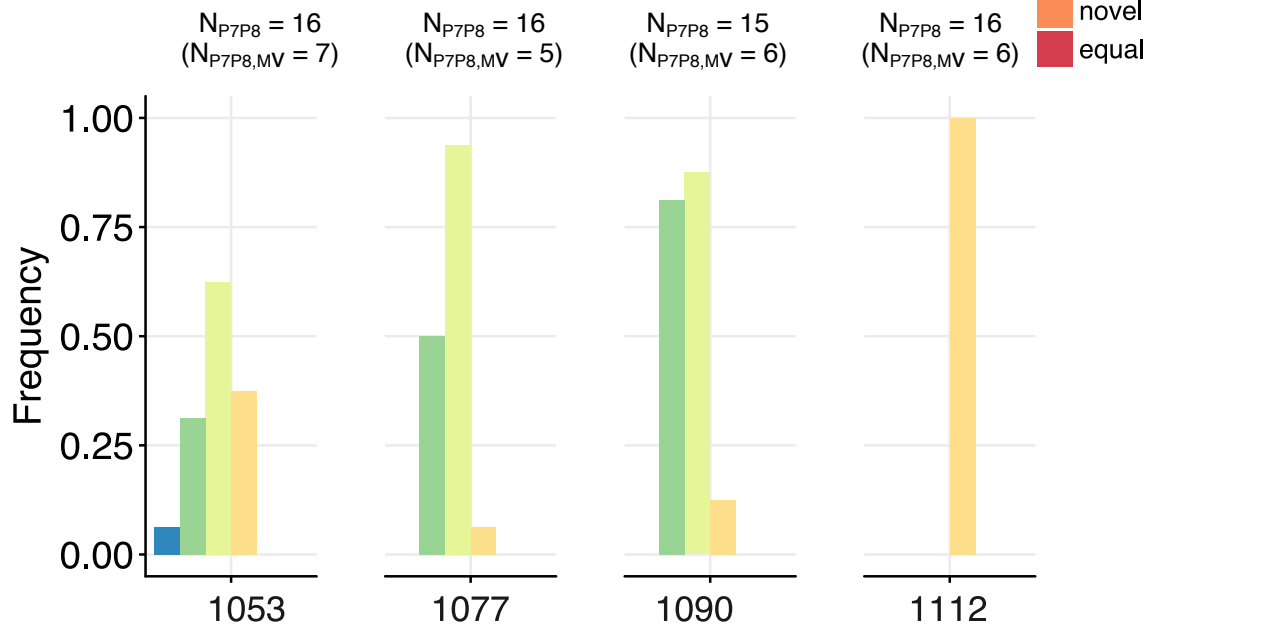

Supplement: Supplementary file 1 — Figure S1. Changes in the total abundance of motifs for specific iKIRs using the sequence based analysis. All HLA-I eluted 9-mers were grouped according to (A) their unique amino acid combinations in P7 and P8 (i.e. assuming the maximal iKIR specificity), or (B) into four distinct groups based on their physico-chemical properties. The number of unique KIR motifs (i.e., unique amino acid pairs, or pairs of amino acids groups in P7 and P8) are given by NP7P8, out of which NP7P8;MV motifs are derived from viral peptides. In this analysis, we monitor for each iKIR motif whether its abundance increases (yellow), or a decreases (lime green) after infection with MV. Using the percentual change, we also quantify the changes in ligand density after MV infection. The frequency of decreases by at least 50% is depicted in dark green and the loss in ligands in blue. Novel ligands, i.e., peptides carrying the KIR binding motifs present only in infected cells are represented in orange. (PDF 90.5 KB) [file 251_2017_1019_MOESM1_ESM.pdf]
